# Mitochondrial GWA Analysis of Lipid Profile Identifies Genetic Variants to Be Associated with HDL Cholesterol and Triglyceride Levels

**DOI:** 10.1371/journal.pone.0126294

**Published:** 2015-05-06

**Authors:** Antònia Flaquer, Susanne Rospleszcz, Eva Reischl, Sonja Zeilinger, Holger Prokisch, Thomas Meitinger, Christa Meisinger, Annette Peters, Melanie Waldenberger, Harald Grallert, Konstantin Strauch

**Affiliations:** 1 Institute of Medical Informatics, Biometry and Epidemiology, Chair of Genetic Epidemiology, Ludwig-Maximilians-Universität, Munich, Germany; 2 Institute of Genetic Epidemiology, Helmholtz Zentrum München—German Research Center for Environmental Health, Neuherberg, Germany; 3 Research Unit of Molecular Epidemiology, Helmholtz Zentrum München—German Research Center for Environmental Health, Neuherberg, Germany; 4 Institute of Human Genetics, Helmholtz Zentrum München—German Research Center for Environmental Health, Neuherberg, Germany; 5 Institute of Epidemiology II, Helmholtz Zentrum München—German Research Center for Environmental Health, Neuherberg, Germany; Chinese Academy of Science, CHINA

## Abstract

It has been suggested that mitochondrial dysfunction has an influence on lipid metabolism. The fact that mitochondrial defects can be accumulated over time as a normal part of aging may explain why cholesterol levels often are altered with age. To test the hypothesis whether mitochondrial variants are associated with lipid profile (total cholesterol, LDL, HDL, and triglycerides) we analyzed a total number of 978 mitochondrial single nucleotide polymorphisms (mtSNPs) in a sample of 2,815 individuals participating in the population-based KORA F4 study. To assess mtSNP association while taking the presence of heteroplasmy into account we used the raw signal intensity values measured on the microarray and applied linear regression. Ten mtSNPs (mt3285, mt3336, mt5285, mt6591, mt6671, mt9163, mt13855, mt13958, mt14000, and mt14580) were significantly associated with HDL cholesterol and one mtSNP (mt15074) with triglycerides levels. These results highlight the importance of the mitochondrial genome among the factors that contribute to the regulation of lipid levels. Focusing on mitochondrial variants may lead to further insights regarding the underlying physiological mechanisms, or even to the development of innovative treatments. Since this is the first mitochondrial genome-wide association analysis (mtGWAS) for lipid profile, further analyses are needed to follow up on the present findings.

## Introduction

Cholesterol is a lipid which is vital for the normal functioning of the body [1]. Having an excessively high level of total cholesterol (TC) itself does not cause any symptoms, but it increases the risk of serious health conditions [2]. Cholesterol is carried around the body in the blood by lipoproteins, in particular Low-density lipoprotein (LDL), High-density lipoprotein (HDL), and very low density lipoprotein (VLDL). LDL carries cholesterol from the liver to the cells. If there is too much cholesterol for the cells to use, it can build up in the artery walls, leading to atherosclerosis [3, 4]. HDL carries cholesterol away from the cells and back to the liver, where it is either broken down or prepared to be excreted from the body as a waste product. High concentrations of HDL particles have protective value against cardiovascular diseases [5]. VLDL contains the highest amount of triglycerides (TG), which have been linked to atherosclerosis and the subsequent risk of heart diseases and stroke [6]. A high TG level combined with a low HDL or high LDL concentration can speed up the process of plaque formation in the arteries resulting in atherosclerosis. The balance of cholesterol levels is important not just for cardiovascular health [7–10] but also for mental health [11, 12]. Control of cholesterol might reduce the brain plaques linked to Alzheimer's disease [13]. It has been also suggested that an excess of cholesterol in mitochondria can result in mitochondrial dysfunction and impairment of specific carriers (e.g. mitochondrial transport of cellular glutathione) through alterations in the mitochondrial membrane order [14–16]. Moreover, previous findings have associated an excessive accumulation of cholesterol in mitochondria with neurodegeneration and myocardial ischemia injury [17, 18] as well as with an increased mitochondrial damage in cardiovascular tissues [19]. An excess production of reactive oxygen species (ROS) in mitochondria, accumulation of mitochondrial DNA (mtDNA) damage, and progressive respiratory chain dysfunction have been related to atherosclerosis [20–23].

The primary function of mitochondria is to generate large quantities of energy in the form of adenosine triphosphate (ATP). mtDNA is of approximately 16.6 kb and codes for 13 genes of the mitochondrial respiratory chain complexes, 2 ribosomal RNA (rRNA) genes, and 22 transfer RNA (tRNA) genes that are required for mitochondrial protein synthesis. Mitochondria consume oxygen and substrates to generate the vast majority of ATP while producing ROS, also called free radicals, in the process. An excess of ROS may damage DNA, proteins, and lipids if not rapidly quenched. This damage, termed oxidative stress, has been suggested to influence cholesterol flux [24, 25].

The 13 structural genes are essential for energy production through the process of oxidative phosphorylation (OXPHOS) that consists of five enzyme complexes (I–V). In addition to supplying cellular energy and involvement in oxidative stress, mitochondria also participate in a wide range of other cellular processes, including signal transduction, cell cycle regulation, thermogenesis, and apoptosis. Mitochondrial mutations can be both somatic and inherited through the maternal line [26]. One peculiarity of mtDNA is the heteroplasmy effect which was originally believed to be a rare phenomenon. Since many mtDNA copies are present in a cell and due to their high mutation rate, new mutations may arise among many of other mtDNA, consequently mutant and wild-type mtDNA can co-exist [27]. For this reason there is heterogeneity of mtDNA within an individual, and even within the same cell. The clinical expression of some phenotypes is determined by the relative proportion of wild-type and mutant mitochondrial genetic variants in different tissues [27]. Variants of mtDNA are under a growing scientific spotlight and there is increasing evidence that these mutations play a central role in many human diseases.

Despite the physiological role of cholesterol in mitochondria, the mechanisms involved in the trafficking to this compartment are poorly understood [28]. Genome-wide association studies and human genetic studies have identified a number of genes and genetic regions affecting cholesterol profile (including TC, HDL, LDL) and TG [7, 8, 10, 29–36]. Nevertheless, none of them have investigated the mitochondrial genome. The purpose of the current study was to conduct a mitochondrial GWAS to identify genetic variants influencing cholesterol phenotypes, including TC, HDL, LDL, and TG. In particular, we tested 978 mtSNPs in a population-based sample of 2,815 adults, aged 31–85 years.

## Methods

### Study design and population

The Cooperative Health Research in the Region of Augsburg (KORA) study is a series of independent population-based epidemiological surveys and follow-up studies of participants living in the region of Augsburg, in southern Germany, an area with demographic and socioeconomic characteristics roughly reflecting those of an average central European population. The study was approved by the local ethics committee (Bayerische Landesärztekammer). All participants are residents of German nationality identified through the registration office and written informed consent was obtained from each participant [37]. The study was approved by the local ethics committee. All participants filled in a self-administrated questionnaire and underwent a standardized personal interview and an extensive medical examination. All procedures were subjected to quality assessment. The study design, sampling method, and data collection have been described in detail elsewhere [38]. The present study includes data of the study KORA F4 (2006–2008) including a total number of 2,815 unrelated individuals. No evidence of population stratification has been found in multiple published analyses using the KORA cohort. Ascertainments of anthropometric measurements and personal interviews, as well as laboratory measurements of persons, from the KORA F4 have been described elsewhere [39]. In order to avoid confounding with insulin-dependent diabetes mellitus, 213 individuals diagnosed with type 2 diabetes were not included in the study, i.e., the 2,815 individuals considered in our analysis do not include persons affected by type 2 diabetes.

### Genotyping and genotype calling

DNA was extracted from full blood after the blood draw and then stored at -80°C. Only single-nucleotide polymorphisms located in the mitochondrial genome (mtSNPs) were considered in this study. Genotyping was performed using the following platforms: Affymetrix 6.0 GeneChip array (465 mtSNPs), Affymetrix Axiom chip array (252 mtSNPs), Illumina Human Exome Beadchip array (226 mtSNPs), and Illumina MetaboChip 200K (135 mtSNPs). The Affymetrix 6.0 chip was genotyped only for a subgroup of 1,814 randomly selected participants of KORA F4. All other chips were genotyped for the whole KORA F4 dataset. The number of individuals used in this analysis corresponds to those that passed genotyping QC, have available phenotype information, and are not affected with type 2 diabetes.

Most of the covered mtSNPs have distinct positions identified by different chips. Although the Affymetrix 6.0 is the one containing the largest number of mtSNPs some regions are not well covered. The Illumina Metabochip contains the smallest number of mtSNPs and many regions are uncovered, especially the hypervariable regions of the mtDNA control region (HVR I and HVR II) as well as the CO1 and CO2 genes. However, when all chips are considered together, good overall coverage of the mitochondrial genome is obtained [40]. Standard genotype calling may be controversial when applied to mtSNPs due to the possible occurrence of heteroplasmy. mtDNA tends to be heterogenous in the sense that different mitochondria of an individual can have different genotypes, such that at an mtSNP may not be restricted to 0, 1, or 2 minor alleles. This issue affects the possibility of estimating genotypes and makes the calling algorithms useless. Therefore, whenever one intends to identify susceptibility genes located in the mtDNA it is recommended to account for heteroplasmy using individual-level allele frequencies obtained from intensity values [40] or sequencing data rather than genotype calls obtained by algorithms that were designed for nuclear SNPs.

### Cholesterol phenotypes (TC, HDL, LDL) and TG

All KORA F4 participants were subjected to several medical examinations including measures of cholesterol phenotypes and fasting TG. TC levels are based on the HDL, LDL and TG levels (TC = HDL + LDL + 0.2 × TG). There are several advantages to analyzing cholesterol phenotypes as a quantitative phenotype in a representative population-based sample of subjects. The quantitative nature of the phenotype increases the power of the study considerably. The use of a general population sample as KORA F4 reduces the number of subjects taking antilipidemic medication compared with patient groups. The distribution of characteristics of the study population is given in Table 1.

**Table 1:** Distribution of characteristics of the study population.

Chip	Affy. 6.0		Affy. Axiom		Illum. Exome		Illum. Metabochip	
**Sample size**	1640		2721		2710		2804	
**Males Females**	**786**	**854**	**1295**	**1426**	**1290**	**1420**	**1334**	**1470**
**Mean age**	60.4±8.8	60.1±8.7	55.6±13.2	55±13	55.5±13.2	54.8±13	55.5±13.2	54.9±13
**Mean TC**	218.5±38.1	228.5±38.7	215±38.6	219.2±39.9	215.1±38.6	218.7±39.8	214.8±38.4	219.8±39.6
**Mean HDL**	51±12.8	62.4±14.4	50.7±12.4	61.8±14.3	50.6±12.5	61.8±14.3	50.6±12.5	61.6±14.3
**Mean LDL**	139±33.7	142.7±35.9	139±33.2	135±35.9	139.1±33.4	134.7±35.8	138.9±33.2	135.1±35.8
**Mean TG**	147.7±108.8	111.8±59.8	140.7±102.9	104.6±59.5	141.6±104.1	103.9±59.4	140.7±103.1	104.8±60.1

Sample size is based on the particular chip. Total sample size is 2,815 independent individuals. One person may be present on more than one chip. Distributions are presented as means ± standard deviation.

### Quality control

Quality control for the signal intensity values was performed for each genotyping chip as described in detail elsewhere [40]. An attempt to remove the chip-specific global background intensity was made by computing, separately for each individual, the 5% quantile intensity and subtracting it from all intensities. In a second step, the intensities were quantile normalized applying the method proposed by Bolstad et al. [41] and implemented in the limma R package [42]. After quantile normalization log_2_ intensity ratios, log_2_($\overset{-}{A}/\overset{-}{B}$), were computed for each individual and an iterative outlier detection procedure was applied [40]. A summary of the quality control results is given in Table 2. From the original number of mtSNPs, 63 (5.8%) were removed because their position could not be placed in Build 38. For the Axiom chip, 37 mtSNPs (17%) were removed due to an upper bound cut-off that has been described in detail in our previous paper [40]. A total number of 498 (<0.05%) intensity ratios were considered to be outliers and removed from the analysis.

**Table 2:** Summary of the quality control.

Chip	mtSNPs	mtSNPs excludedUB no_B38		Sample size	I_SNP_	I_tot_	Intensity RatioOutliers
Affy. 6.0	**411**	0	54	1,647	3	4,061,502	230 (<0.05%)
Affy. Axiom	**215**	37	0	2,731	4	4,697,320	42 (<0.05%)
Illum. Exome	**226**	0	0	2,721	1	1,229,892	128 (<0.05%)
Illum. Metabo	**126**	0	9	2,815	1	709,380	98 (<0.05%)

The number of **mtSNPs** refers to the SNPs that passed QC and were included in the analysis. Several mtSNPs were excluded due to the upper bound cut-off (**UB**) [77] or because the basepair position was not available in Build 38 (**no_B38**). Sample size is based on the particular chip. Total sample size is 2,803 independent individuals. One person may be present on more than one chip. *I*_*SNP*_ stands for the number of intensity measures per allele. *I*_*tot*_ represents the total number of intensity measures in the sample (I_SNP_*2*sample-size*mtSNPs).

### Statistical method

To approach the presence of heteroplasmy present in the mitochondria we used the raw signals of luminous intensity, where every measurement is associated with a specific mtSNP and represents one of its alleles. The number of measures *n* per mtSNP depends on the vendor-specific technology employed on the genotyping chip. To assess association of cholesterol phenotypes with the mtSNPs intensities we applied linear regression analysis using cholesterol levels as outcome. The mtSNP enters the model as a covariate via the log_2_-transformed intensity ratio, log_2_($\overset{-}{A}/\overset{-}{B}$), where $\overset{-}{A}\;and\;\overset{-}{B}$ denote the mean intensity over *n* measures, or single measure in case of *n* = 1, for the A allele and B allele (minor allele), respectively. We center this variable (z = log_2_($\overset{-}{A}/\overset{-}{B}$)-μ) as well as the additional quantitative covariate age at examination, to improve the convergence properties of the model estimates. Sex is also introduced in the model as covariate with male as a baseline. Each type of genotyping chip needs to be analyzed separately because different chips make use of different technologies, even between chips of the same manufacturer. In each of the analyses p-values are obtained from a Wald test and adjusted for multiple comparisons applying the Bonferroni correction method with the correction factor being equal to the number of mtSNPs used in the analysis. All the analyses were performed with the statistical software R v3.1.0 [43]. For more details about the statistical method we refer to [40].

## Results

**Fig 1:**
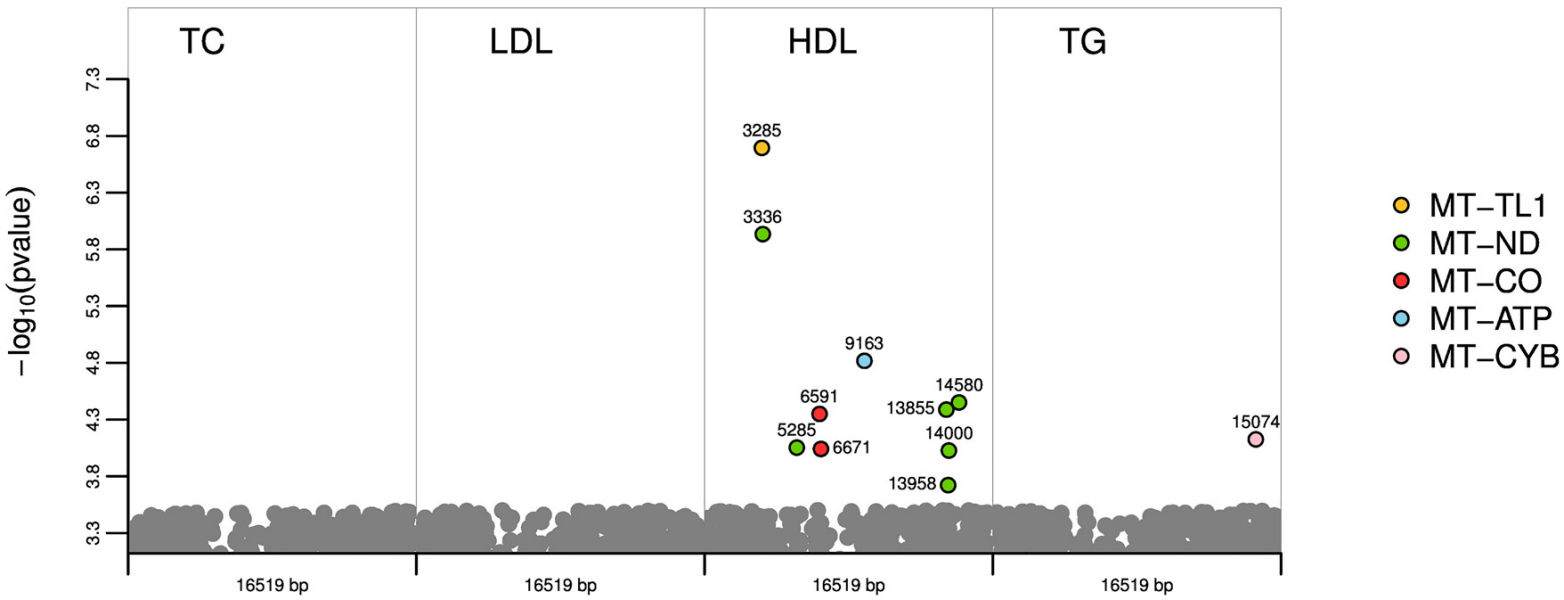
Illustration by phenotype of the 11 significant mtSNPs after correcting for multiple testing. On the y axis, the p-values transformed into the negative of the base 10 logarithm, −log_10_(p-value), are shown. The x-axis represents the mitochondrial genome for each phenotype.

After QC, a total number of 978 mtSNPs were included in the analysis. The resulting significant p-values are plotted in Fig 1 for each phenotype. A more detailed figure for each genotyping chip and phenotype is provided in S1 Fig. No significant mtSNPs were obtained for TC. However, when analysing cholesterol subtypes, ten mtSNPs for HDL cholesterol and one mtSNP for TG reached significance after correcting for multiple testing. The association results that remained significant after adjustment for multiple testing (P_adjusted_ ≤ 0.05) are presented in Table 3. Some of the significant mtSNPs from the Affymetrix chip (mt3336, mt5285, and mt14000) were also present in other chips. Although these variants also resulted to be nominally significant when analysing the other chips, they lost their significance after adjusting for multiple testing (see Table 3).

**Table 3:** Summary of significant mtSNPs.

Chip	Bp rs_number	Alleles (maf)	Point mutation	β_SNP_	P_nominal_ (P_adj_)	Overlap Chip: P_nominal_	Protein: *Gene*
**HDL**							
Affy.6.0	3285 rs28537613	T→A (n.a.)	-	3.49	2.2x10^-07^ (8.3x10^-05^)	-	tRNA^Leu(UUR)^: *MT-TL1*
Affy.6.0	3336 rs28416101	T→G (0.0033)	missense	0.91	1.2x10^-06^ (4.8x10^-04^)	Axiom: 0.022	ND1: *MT-ND1*; subunit of NADH dehydrogenase, complex I
Affy.6.0	5285 rs28357986	A→G (0.0030)	synonymous	-4.63	8.9x10^-05^ (3.6x10^-02^)	Axiom: 0.013	ND2: *MT-ND2;* subunit of NADH dehydrogenase, complex I
Affy.6.0	6591 rs28483589	C→A (n.a.)	missense	-3.09	4.5x10^-05^ (1.8x10^-02^)	-	COI: *gene MT-CO1;* subunit of cytochrome c oxidase, complex IV
Affy.6.0	6671 rs1978028	T→C (0.0189)	synonymous	-2.09	9.1x10^-05^ (3.7x10^-2^)	-	COI: *gene MT-CO1;* subunit of cytochrome c oxidase, complex IV
Affy.6.0	9163 rs2298010	G→A (0.0004)	missense	4.47	1.5x10^-05^ (6.3x10^-03^)	-	ATP6: *MT-ATP6;* subunit of ATP synthase, complex V
Affy.6.0	13855 rs3925298	C→T (0.0011)	synonymous	-5.19	4.1x10^-05^ (1.7x10^-2^)	-	ND5: *MT-ND5;* subunit of NADH dehydrogenase, complex I
Illum. Exome	13958 rs202081448	G→C (0.0037)	missense	-2.32	1.90x10^-04^ (4.2x10^-2^)	-	ND5: *MT-ND5;* subunit of NADH dehydrogenase, complex I
Affy.6.0	14000 rs28359185	T→A (0.0100)	missense	4.48	9.4x10^-05^ (3.9x10^-2^)	Axiom: 8.3x10^-03^ Exome: 2.1x10^-03^ Metabo: 1.0x10^-03^	ND5: *MT-ND5;* subunit of NADH dehydrogenase, complex I
Affy 6.0	14580 rs28496897	A→G (0.0004)	synonymous	1.87	3.5x10^-05^ (1.4x10^-02^)	-	ND6: *MT-ND6;* subunit of NADH dehydrogenase, complex I
**Triglycerides**							
Illum. Exome	15074 rs201169089	T→C (n.a.)	missense	-14.9	7.5x10^-05^ (1.6x10^-02^)	-	CYTB: *MT-CYB*, cytochrome c reductase, complex III

Genomic position in base pairs (bp), alleles, rs_number, and point mutation are based on the NCBI dbSNP GRCh38 human genome assembly (rCRS, GeneBank ID J01415.2). Alleles are given in terms of major→minor allele. The population minor allele frequency “maf” is based on 2,704 individuals provided by mitomap (http://www.mitomap.org). Note that these allele frequency estimates do not account for the presence of heteroplasmy. An estimated effect size (β_SNP_) < 0 indicates that the risk allele is the minor allele. Nominal p-values and adjusted p-values are provided. mtSNPs mt3336, mt5285 and mt14000 are also included in other chips.

A negative parameter estimate for the mtSNP (β_SNP_ < 0) indicates that the minor allele is associated with an increase of the level of cholesterol subtype while a β_SNP_ > 0 indicates that the major allele is associated with high levels of cholesterol subtype.

### TG levels

For TG levels the only significant mtSNP was found in the *MT-CYB* gene (mt15074T→C). The presence of mt15074T→C heteroplasmy with more C than T alleles results in a higher level of TG than having only T alleles (β_mt15074T→C_ = -14.9). In the regression model for this mtSNP the estimates of sex and age were both significant with β_sex_ < 0 and β_age_ >0, indicating significantly higher TG levels in males than in females and with older age.

### HDL cholesterol

Six of the ten significant mtSNPs for HDL cholesterol (mt3336T→G, mt5285A→G, mt13855C→T, mt13958G→C, mt14000T→A, and mt14580A→G) are located in the NADH subunit dehydrogenase genes of complex I (*MT-ND1*, *MT-ND2*, *MT-ND5*, and *MT-ND6*), the others are located in the ATP synthase subunit 6 (*MT-ATP6*), cytochrome c oxidase subunit 1 (*MT-CO1*),and tRNA (*MT-TL1*).

Taking the most strongly associated variant mt3285T→A, in the tRNA, *MT-TL1* gene, based on our estimates (β_mt3285T→A_ = 3.49) HDL cholesterol increases with a higher proportion of T alleles at this locus, making the T allele favourable to HDL. Similar arguments can be applied to the other mtSNPs. The regression models for all significant mtSNPs regarding HDL showed no significant effect for age; however, the estimate of β_sex_ was significant with β_sex_>0 indicating significantly higher levels of HDL cholesterol in females than in males.

## Discussion

We performed a mitochondrial GWA analysis for the lipid profile including TC, TG, HDL cholesterol, and LDL cholesterol. Ten mtSNPs were significant for HDL and one mtSNP for TG. No significant results were observed for TC and LDL cholesterol. The possible role of mitochondria in the regulation of the lipid profile is mainly concerned with ROS production [24, 25].

### Triglycerides

***MT-CYB***_***mt15074T→C***_ **: *Mitochondrially encoded cytochrome b*, *complex III*,** catalyzes the transfer of electrons from ubiquinol to cytochrome c and utilizes the energy to translocate protons from inside the mitochondrial inner membrane to outside. Complex I and complex III are considered as the major ROS sources [44]. It has been shown that inhibition of complex III trigger the accumulation of TG in 3T3-L1 cells [45, 46]. Mutations of the MT-CYB gene have also been related to exercise intolerance [47–49]. Recently, association of MT-CYB_15059G→A_ mutation heteroplasmy with essential hypertension has been suggested [50].

Oxidative stress may also result from the metabolic impact of intracellular TG. Lipids and glucose that are not needed for immediate use are stored in adipose tissues and liver in the form of TG in lipid droplets. It has been demonstrated that hyperglycemia (excess of glucose) induces production of ROS [51, 52], which further causes partial inhibition of the electron transport in complex III [52].

The estimates of the regression model with *MT-CYB*_*mt15074T→C*_ variant are in line with the fact that males have higher TG levels than females [53, 54] and increase with older age since as people age and gain weight, TG levels generally increase.

### HDL cholesterol

Among the ten mtSNPs identified in this study for HDL cholesterol six are located in NADH genes, the rest are located in the *MT-TL1*, *MT-ATP6*, *and MT-CO1*.

***MT-ND1***_**mt3336T→G**_ **, *MT-ND2***_**mt5285A→G**_ **, *MT-ND5***_**mt13855C→T, mt13958G→C, mt14000T→A**_ **, and *MT-ND6***_**mt14580A→G**_ **: *Mitochondrially encoded NADH dehydrogenase subunits*, *complex I*,** extracts energy from NADH, produced by the oxidation of sugars and lipids, and traps the energy in a potential difference or voltage across the mitochondrial inner membrane. The potential difference is used to power the synthesis of ATP. Because complex I is central to energy production in the cell, its malfunction may result in a wide range of disorders. Some of them are due to mutations, while others, which result from a decrease in the activity of complex I, or an increase in the production of ROS, are not yet well understood. Despite the fact that one study demonstrated that the polymorphism *MT-ND2*_mt5178A→C_ is associated with HDL-C levels in Japanese subjects [55], very little has been reported about relationships between NADH subunits and HDL.

***MT-TL1***_**mt3285T→A**_ **: *Mitochondrially encoded tRNA leucine 1 gene (MT-TL1)*** provides instructions for making a specific form of tRNA that is designated as tRNA^Leu(UUR)^. Mutations of the MT-TL1 gene may play a pathogenic role in the formation of atherosclerotic lesions of human arteries, causing various defects in the protein chains of some tRNA, synthesized directly in the mitochondria. This leads to a decrease in the concentration of these enzymes and their tRNA or total dysfunction, which contributes to the development of oxidative stress and increases the probability of occurrence and development of atherosclerosis [56]. Particularly the variant *MT-TL1*_mt3256C→T_ has been related to atherosclerosis predisposition [57]. There is unequivocal evidence of an inverse association between plasma HDL cholesterol concentrations and the risk of cardiovascular disease, a finding that has led to the hypothesis that HDL protects from atherosclerosis [58].

***MT-ATP6***_***mt9163G→A***_ **: *Mitochondrially encoded ATP synthase*, *complex V*,** is an important enzyme that produces most of cellular ATP. Alteration of ATP synthase biogenesis may cause two types of isolated defects: either the enzyme is structurally modified and does not function properly, or it is present in abnormal amounts. In both cases the cellular energy provision is impaired, which leads to a dysregulation of ROS production [59]. The presence of two principal proteins of the mitochondrial ATP synthase, β-chain and α-chain, on the surface of human hepatocytes have been associated with HDL catabolism for the control of cholesterolemia [60, 61]. However, how the cell directs these proteins towards the cell surface and how their cell-surface expression is regulated remain unknown and require further investigation.

***MT-CO1***_**mt6591C→A, mt6671T→C**_ **: *Mitochondrially encoded cytochrome c oxidase subunits*, *complex IV*,** is a key oxidative enzyme regarded as one of the major regulation sites for the OXPHOS system, controlled by both nDNA and mtDNA. Its catalytic activity is primarily determined by three of the 13 subunits which are encoded by the mtDNA (*MT-CO1*, *MT-CO2*, and *MT-CO3*) [62]. The loss of function of this enzyme has been suggested to trigger ROS production, although the increase in radical accumulation rests with non-mitochondrial sources [63]. However, the function of each subunit and the molecular mechanism behind the regulation of the activity of this important protein complex are largely unknown [64]. Although a direct relationship between HDL and variants in the *MT-CO1* genes has not been reported yet, a significant inverse correlation between the hepatic *MT-CO1* methylated/unmethylated DNA ratio and HDL has been observed [65].

The regression models for all significant mtSNPs identified for HDL cholesterol in this study also corroborate the generally acknowledged fact that females have significantly higher HDL cholesterol values than males [66–68]. Progesterone, anabolic steroids and male sex hormones (testosterone) also lower HDL cholesterol levels while female sex hormones raise HDL cholesterol levels. Age was not significant in our study, meaning that HDL cholesterol levels do not vary with age, a finding also reported from other studies [69, 70]. However TC and LDL cholesterol levels tend to vary with age [70, 71].

It has been suggested that HDL potentially inhibits apoptosis in endothelial cells [72–74]. This effect is paralleled by decreased intracellular generation of ROS and diminished levels of apoptotic markers, suggesting that it can be related to the intracellular antioxidative actions of HDL or HDL components. HDL is also able to inhibit generation of ROS in vitro under conditions of cell culture [74, 75].

Some of the variants identified in this study are missense mutations which lead to an amino acid change, thus being a non-synonymous variant. So, individuals with an excess of missense mutations may carry an appreciable fraction of an altered protein that is responsible for altering the levels of the phenotype. Other variants are synonymous, i.e., they code for the same amino acid. How an excess of synonymous mutations at this locus could impact the levels of HDL cholesterol needs further investigation, since the single nucleotide change leads to an unchanged protein. However, different codons might lead to different protein expression levels. Based on these findings we hypothesize that levels of HDL and TG are attributable at least in part to mitochondrial polymorphisms. Animal and human data consistently show that mitochondria are altered in aging, leading to increased mutations in mtDNA, decreased expression of some mitochondrial proteins, reduced enzyme activity, and altered respiration with reduced maximal capacity in sedentary adults. The possible role of mitochondria in the regulation of HDL cholesterol and TG is mainly concerned with ROS production. However, the complexity of mitochondrial ROS metabolism suggests that interventions such as the administration of one or a few antioxidants may be too simplistic. A more complete approach to antioxidant therapy might be to decrease ROS generation (for example, by expressing uncoupling proteins) and to upregulate the multilayered endogenous mitochondrial and intracellular antioxidant defense network [76]. However, this will require a considerably better understanding of ROS biology than we have at present

## Conclusions

In summary, our study reports eleven mitochondrial genetic variants, ten of which are significantly associated with HDL cholesterol and one with TG levels, indicating that the presence of heteroplasmy in these variants may influence the balance of HDL cholesterol and TG levels. Although further analyses are needed to follow up on the present results, these findings highlight the important role of the mtDNA among the factors that contribute to the balance of the lipid profile in adults and suggest that variants in the mitochondrial genome may be more important than has previously been suspected.

## Supporting Information

S1 Fig Mitochondrial genome-wide *P* values by chip and phenotype. On the y axis, p-values transformed into the negative of the base 10 logarithm, −log10(p-value), are shown. The x-axis represents the mitochondrial genome, displaying the position and relative size of each of the 13 major mitochondrial genes, 12S and 16S rRNAs, hypervariable region 1 (HVR I), hypervariable region 2 (HVR II) as well as the position of the 22 tRNAs (gray). The left side illustrates the results for TC and the right side illustrates the results for cholesterol subtypes (LDL, HDL, and triglycerides). The dashed lines show the critical values of the pointwise significance level corresponding to α = 0.05.
(TIFF)

## References

[1] National Institutes of Health MP. Cholesterol Bethesda, US 3 2014 10.1155/2014/109263

[2] Lee CH, Olson P, Evans RM. Minireview: lipid metabolism, metabolic diseases, and peroxisome proliferator-activated receptors. Endocrinology. 2003;144(6):2201–7. 10.1210/en.2003-0288 . 12746275

[3] Brown MS, Goldstein JL. How LDL receptors influence cholesterol and atherosclerosis. Scientific American. 1984;251(5):58–66. . 6390676 10.1038/scientificamerican1184-58

[4] Ravnskov U. Is atherosclerosis caused by high cholesterol? QJM: monthly journal of the Association of Physicians. 2002;95(6):397–403. . 12037248 10.1093/qjmed/95.6.397

[5] Toth PP. Cardiology patient page. The "good cholesterol": high-density lipoprotein. Circulation. 2005;111(5):e89–91. 10.1161/01.CIR.0000154555.07002.CA . 15699268

[6] Talayero BG, Sacks FM. The role of triglycerides in atherosclerosis. Current cardiology reports. 2011;13(6):544–52. 10.1007/s11886-011-0220-3 21968696 PMC3234107

[7] Aulchenko YS, Ripatti S, Lindqvist I, Boomsma D, Heid IM, Pramstaller PP, et al Loci influencing lipid levels and coronary heart disease risk in 16 European population cohorts. Nature genetics. 2009;41(1):47–55. 10.1038/ng.269 19060911 PMC2687074

[8] Willer CJ, Sanna S, Jackson AU, Scuteri A, Bonnycastle LL, Clarke R, et al Newly identified loci that influence lipid concentrations and risk of coronary artery disease. Nature genetics. 2008;40(2):161–9. 10.1038/ng.76 . 18193043 PMC5206900

[9] Mangiacapra F, De Bruyne B, Peace AJ, Melikian N, Wijns W, Barbato E. High cholesterol levels are associated with coronary microvascular dysfunction. Journal of cardiovascular medicine. 2012;13(7):439–42. 10.2459/JCM.0b013e328351725a . 22343265

[10] Wallace C, Newhouse SJ, Braund P, Zhang F, Tobin M, Falchi M, et al Genome-wide association study identifies genes for biomarkers of cardiovascular disease: serum urate and dyslipidemia. American journal of human genetics. 2008;82(1):139–49. 10.1016/j.ajhg.2007.11.001 18179892 PMC2253977

[11] West R, Beeri MS, Schmeidler J, Hannigan CM, Angelo G, Grossman HT, et al Better memory functioning associated with higher total and low-density lipoprotein cholesterol levels in very elderly subjects without the apolipoprotein e4 allele. The American journal of geriatric psychiatry: official journal of the American Association for Geriatric Psychiatry. 2008;16(9):781–5. 10.1097/JGP.0b013e3181812790 18757771 PMC2614555

[12] Saher G, Brugger B, Lappe-Siefke C, Mobius W, Tozawa R, Wehr MC, et al High cholesterol level is essential for myelin membrane growth. Nature neuroscience. 2005;8(4):468–75. 10.1038/nn1426 . 15793579

[13] Reed B, Villeneuve S, Mack W, DeCarli C, Chui HC, Jagust W. Associations between serum cholesterol levels and cerebral amyloidosis. JAMA neurology. 2014;71(2):195–200. 10.1001/jamaneurol.2013.5390 . 24378418 PMC4083819

[14] Colell A, Garcia-Ruiz C, Lluis JM, Coll O, Mari M, Fernandez-Checa JC. Cholesterol impairs the adenine nucleotide translocator-mediated mitochondrial permeability transition through altered membrane fluidity. The Journal of biological chemistry. 2003;278(36):33928–35. 10.1074/jbc.M210943200 . 12821666

[15] Lluis JM, Colell A, Garcia-Ruiz C, Kaplowitz N, Fernandez-Checa JC. Acetaldehyde impairs mitochondrial glutathione transport in HepG2 cells through endoplasmic reticulum stress. Gastroenterology. 2003;124(3):708–24. 10.1053/gast.2003.50089 . 12612910

[16] Fernandez-Checa JC, Kaplowitz N. Hepatic mitochondrial glutathione: transport and role in disease and toxicity. Toxicology and applied pharmacology. 2005;204(3):263–73. 10.1016/j.taap.2004.10.001 . 15845418

[17] Rouslin W, MacGee J, Gupte S, Wesselman A, Epps DE. Mitochondrial cholesterol content and membrane properties in porcine myocardial ischemia. The American journal of physiology. 1982;242(2):H254–9. . 6461257 10.1152/ajpheart.1982.242.2.H254

[18] Yu W, Gong JS, Ko M, Garver WS, Yanagisawa K, Michikawa M. Altered cholesterol metabolism in Niemann-Pick type C1 mouse brains affects mitochondrial function. The Journal of biological chemistry. 2005;280(12):11731–9. 10.1074/jbc.M412898200 . 15644330

[19] Knight-Lozano CA, Young CG, Burow DL, Hu ZY, Uyeminami D, Pinkerton KE, et al Cigarette smoke exposure and hypercholesterolemia increase mitochondrial damage in cardiovascular tissues. Circulation. 2002;105(7):849–54. . 11854126 10.1161/hc0702.103977

[20] Madamanchi NR, Runge MS. Mitochondrial dysfunction in atherosclerosis. Circulation research. 2007;100(4):460–73. 10.1161/01.RES.0000258450.44413.96 . 17332437

[21] Puddu P, Puddu GM, Cravero E, De Pascalis S, Muscari A. The emerging role of cardiovascular risk factor-induced mitochondrial dysfunction in atherogenesis. Journal of biomedical science. 2009;16:112 10.1186/1423-0127-16-112 20003216 PMC2800844

[22] Chang JC, Kou SJ, Lin WT, Liu CS. Regulatory role of mitochondria in oxidative stress and atherosclerosis. World journal of cardiology. 2010;2(6):150–9. 10.4330/wjc.v2.i6.150 21160733 PMC2999054

[23] Mercer JR, Cheng KK, Figg N, Gorenne I, Mahmoudi M, Griffin J, et al DNA damage links mitochondrial dysfunction to atherosclerosis and the metabolic syndrome. Circulation research. 2010;107(8):1021–31. 10.1161/CIRCRESAHA.110.218966 20705925 PMC2982998

[24] Gesquiere L, Loreau N, Minnich A, Davignon J, Blache D. Oxidative stress leads to cholesterol accumulation in vascular smooth muscle cells. Free radical biology & medicine. 1999;27(1–2):134–45. . 10443930 10.1016/s0891-5849(99)00055-6

[25] Marcil V, Delvin E, Sane AT, Tremblay A, Levy E. Oxidative stress influences cholesterol efflux in THP-1 macrophages: role of ATP-binding cassette A1 and nuclear factors. Cardiovascular research. 2006;72(3):473–82. 10.1016/j.cardiores.2006.08.024 . 17070507

[26] Giles RE, Blanc H, Cann HM, Wallace DC. Maternal inheritance of human mitochondrial DNA. Proceedings of the National Academy of Sciences of the United States of America. 1980;77(11):6715–9. 6256757 10.1073/pnas.77.11.6715 PMC350359

[27] Wallace DC, Chalkia D. Mitochondrial DNA genetics and the heteroplasmy conundrum in evolution and disease. Cold Spring Harbor perspectives in medicine. 2013;3(10):a021220 . 10.1101/cshperspect.a021220 PMC3809581 24186072

[28] Garcia-Ruiz C, Mari M, Colell A, Morales A, Caballero F, Montero J, et al Mitochondrial cholesterol in health and disease. Histology and histopathology. 2009;24(1):117–32. . 19012251 10.14670/HH-24.117

[29] Kathiresan S, Manning AK, Demissie S, D'Agostino RB, Surti A, Guiducci C, et al A genome-wide association study for blood lipid phenotypes in the Framingham Heart Study. BMC medical genetics. 2007;8 Suppl 1:S17 10.1186/1471-2350-8-S1-S17 17903299 PMC1995614

[30] Schadt EE, Molony C, Chudin E, Hao K, Yang X, Lum PY, et al Mapping the genetic architecture of gene expression in human liver. PLoS biology. 2008;6(5):e107 10.1371/journal.pbio.0060107 18462017 PMC2365981

[31] Kathiresan S, Melander O, Guiducci C, Surti A, Burtt NP, Rieder MJ, et al Six new loci associated with blood low-density lipoprotein cholesterol, high-density lipoprotein cholesterol or triglycerides in humans. Nature genetics. 2008;40(2):189–97. 10.1038/ng.75 18193044 PMC2682493

[32] Kathiresan S, Willer CJ, Peloso GM, Demissie S, Musunuru K, Schadt EE, et al Common variants at 30 loci contribute to polygenic dyslipidemia. Nature genetics. 2009;41(1):56–65. 10.1038/ng.291 19060906 PMC2881676

[33] Samani NJ, Braund PS, Erdmann J, Gotz A, Tomaszewski M, Linsel-Nitschke P, et al The novel genetic variant predisposing to coronary artery disease in the region of the PSRC1 and CELSR2 genes on chromosome 1 associates with serum cholesterol. Journal of molecular medicine. 2008;86(11):1233–41. 10.1007/s00109-008-0387-2 . 18649068

[34] Nakayama K, Bayasgalan T, Yamanaka K, Kumada M, Gotoh T, Utsumi N, et al Large scale replication analysis of loci associated with lipid concentrations in a Japanese population. Journal of medical genetics. 2009;46(6):370–4. 10.1136/jmg.2008.064063 . 19487539

[35] Sandhu MS, Waterworth DM, Debenham SL, Wheeler E, Papadakis K, Zhao JH, et al LDL-cholesterol concentrations: a genome-wide association study. Lancet. 2008;371(9611):483–91. 10.1016/S0140-6736(08)60208-1 18262040 PMC2292820

[36] Global Lipids Genetics C, Willer CJ, Schmidt EM, Sengupta S, Peloso GM, Gustafsson S, et al Discovery and refinement of loci associated with lipid levels. Nature genetics. 2013;45(11):1274–83. 10.1038/ng.2797 24097068 PMC3838666

[37] Wichmann HE, Gieger C, Illig T. KORA-gen—resource for population genetics, controls and a broad spectrum of disease phenotypes. Gesundheitswesen. 2005;67 Suppl 1:S26–30. Epub 2005/07/21. 10.1055/s-2005-858226 . 16032514

[38] Holle R, Happich M, Lowel H, Wichmann HE. KORA—a research platform for population based health research. Gesundheitswesen. 2005;67 Suppl 1:S19–25. Epub 2005/07/21. 10.1055/s-2005-858235 . 16032513

[39] Meisinger C, Strassburger K, Heier M, Thorand B, Baumeister SE, Giani G, et al Prevalence of undiagnosed diabetes and impaired glucose regulation in 35–59-year-old individuals in Southern Germany: the KORA F4 Study. Diabetic medicine: a journal of the British Diabetic Association. 2010;27(3):360–2. 10.1111/j.1464-5491.2009.02905.x . 20536501

[40] Flaquer A, Baumbach C, Kriebel J, Meitinger T, Peters A, Waldenberger M, et al Mitochondrial Genetic Variants Identified to Be Associated with BMI in Adults. PloS one. 2014;9(8):e105116 10.1371/journal.pone.0105116 . 25153900 PMC4143221

[41] Bolstad BM, Irizarry RA, Astrand M, Speed TP. A comparison of normalization methods for high density oligonucleotide array data based on variance and bias. Bioinformatics. 2003;19(2):185–93. Epub 2003/01/23. . 12538238 10.1093/bioinformatics/19.2.185

[42] Smyth GK. limma: Linear Models for Microarray Data In: Gentleman R, editor. Bioinformatics and computational biology solutions using R and Bioconductor Statistics for biology and health. New York: Springer Science+Business Media; 2005 p. 397–420.

[43] R Core Team. R: A Language and Environment for Statistical Computing. Vienna, Austria: R Foundation for Statistical Computing; 2013 10.3758/s13428-013-0330-5

[44] Jezek P, Hlavata L. Mitochondria in homeostasis of reactive oxygen species in cell, tissues, and organism. The international journal of biochemistry & cell biology. 2005;37(12):2478–503. 10.1016/j.biocel.2005.05.013 . 16103002

[45] Vankoningsloo S, Piens M, Lecocq C, Gilson A, De Pauw A, Renard P, et al Mitochondrial dysfunction induces triglyceride accumulation in 3T3-L1 cells: role of fatty acid beta-oxidation and glucose. Journal of lipid research. 2005;46(6):1133–49. 10.1194/jlr.M400464-JLR200 . 15741651

[46] Vankoningsloo S, De Pauw A, Houbion A, Tejerina S, Demazy C, de Longueville F, et al CREB activation induced by mitochondrial dysfunction triggers triglyceride accumulation in 3T3-L1 preadipocytes. Journal of cell science. 2006;119(Pt 7):1266–82. 10.1242/jcs.02848 . 16537646

[47] Legros F, Chatzoglou E, Frachon P, Ogier De Baulny H, Laforet P, Jardel C, et al Functional characterization of novel mutations in the human cytochrome b gene. European journal of human genetics: EJHG. 2001;9(7):510–8. 10.1038/sj.ejhg.5200678 . 11464242

[48] Schuelke M, Krude H, Finckh B, Mayatepek E, Janssen A, Schmelz M, et al Septo-optic dysplasia associated with a new mitochondrial cytochrome b mutation. Annals of neurology. 2002;51(3):388–92. . 11891837 10.1002/ana.10151

[49] Andreu AL, Hanna MG, Reichmann H, Bruno C, Penn AS, Tanji K, et al Exercise intolerance due to mutations in the cytochrome b gene of mitochondrial DNA. The New England journal of medicine. 1999;341(14):1037–44. 10.1056/NEJM199909303411404 . 10502593

[50] Sobenin IA, Chistiakov DA, Sazonova MA, Ivanova MM, Bobryshev YV, Orekhov AN, et al Association of the level of heteroplasmy of the 15059G>A mutation in the MT-CYB mitochondrial gene with essential hypertension. World journal of cardiology. 2013;5(5):132–40. 10.4330/wjc.v5.i5.132 23710300 PMC3663127

[51] Kiritoshi S, Nishikawa T, Sonoda K, Kukidome D, Senokuchi T, Matsuo T, et al Reactive oxygen species from mitochondria induce cyclooxygenase-2 gene expression in human mesangial cells: potential role in diabetic nephropathy. Diabetes. 2003;52(10):2570–7. . 14514642 10.2337/diabetes.52.10.2570

[52] Rolo AP, Palmeira CM. Diabetes and mitochondrial function: role of hyperglycemia and oxidative stress. Toxicology and applied pharmacology. 2006;212(2):167–78. 10.1016/j.taap.2006.01.003 . 16490224

[53] Acarturk E, Cayli M, Akpinar O, Attila G, Demir M. Relation between age and gender differences in plasma triglyceride concentrations and coronary artery disease in Southern Turkey. Clinica chimica acta; international journal of clinical chemistry. 2004;339(1–2):123–8. . 14687902 10.1016/j.cccn.2003.10.001

[54] Seidell JC, Cigolini M, Charzewska J, Ellsinger BM, Bjorntorp P, Hautvast JG, et al Fat distribution and gender differences in serum lipids in men and women from four European communities. Atherosclerosis. 1991;87(2–3):203–10. . 1854366 10.1016/0021-9150(91)90022-u

[55] Kokaze A, Ishikawa M, Matsunaga N, Yoshida M, Sekine Y, Teruya K, et al Association of the mitochondrial DNA 5178 A/C polymorphism with serum lipid levels in the Japanese population. Human genetics. 2001;109(5):521–5. 10.1007/s004390100602 . 11735027

[56] Chistiakov DA, Sobenin IA, Bobryshev YV, Orekhov AN. Mitochondrial dysfunction and mitochondrial DNA mutations in atherosclerotic complications in diabetes. World journal of cardiology. 2012;4(5):148–56. 10.4330/wjc.v4.i5.148 22655163 PMC3364501

[57] Sobenin IA, Sazonova MA, Ivanova MM, Zhelankin AV, Myasoedova VA, Postnov AY, et al Mutation C3256T of mitochondrial genome in white blood cells: novel genetic marker of atherosclerosis and coronary heart disease. PloS one. 2012;7(10):e46573 10.1371/journal.pone.0046573 23056349 PMC3462756

[58] Vergeer M, Holleboom AG, Kastelein JJ, Kuivenhoven JA. The HDL hypothesis: does high-density lipoprotein protect from atherosclerosis? Journal of lipid research. 2010;51(8):2058–73. 10.1194/jlr.R001610 20371550 PMC2903818

[59] Houstek J, Pickova A, Vojtiskova A, Mracek T, Pecina P, Jesina P. Mitochondrial diseases and genetic defects of ATP synthase. Biochimica et biophysica acta. 2006;1757(9–10):1400–5. 10.1016/j.bbabio.2006.04.006 . 16730639

[60] Martinez LO, Jacquet S, Esteve JP, Rolland C, Cabezon E, Champagne E, et al Ectopic beta-chain of ATP synthase is an apolipoprotein A-I receptor in hepatic HDL endocytosis. Nature. 2003;421(6918):75–9. 10.1038/nature01250 . 12511957

[61] Giorgio V, Bisetto E, Franca R, Harris DA, Passamonti S, Lippe G. The ectopic F(O)F(1) ATP synthase of rat liver is modulated in acute cholestasis by the inhibitor protein IF1. Journal of bioenergetics and biomembranes. 2010;42(2):117–23. 10.1007/s10863-010-9270-2 . 20180002

[62] Capaldi RA. Structure and function of cytochrome c oxidase. Annual review of biochemistry. 1990;59:569–96. Epub 1990/01/01. 10.1146/annurev.bi.59.070190.003033 . 2165384

[63] Leadsham JE, Sanders G, Giannaki S, Bastow EL, Hutton R, Naeimi WR, et al Loss of cytochrome c oxidase promotes RAS-dependent ROS production from the ER resident NADPH oxidase, Yno1p, in yeast. Cell metabolism. 2013;18(2):279–86. Epub 2013/08/13. 10.1016/j.cmet.2013.07.005 . 23931758

[64] Li Y, Park JS, Deng JH, Bai Y. Cytochrome c oxidase subunit IV is essential for assembly and respiratory function of the enzyme complex. Journal of bioenergetics and biomembranes. 2006;38(5–6):283–91. Epub 2006/11/09. 10.1007/s10863-006-9052-z 17091399 PMC1885940

[65] Pirola CJ, Gianotti TF, Burgueno AL, Rey-Funes M, Loidl CF, Mallardi P, et al Epigenetic modification of liver mitochondrial DNA is associated with histological severity of nonalcoholic fatty liver disease. Gut. 2013;62(9):1356–63. 10.1136/gutjnl-2012-302962 . 22879518

[66] McNamara JR, Campos H, Ordovas JM, Peterson J, Wilson PW, Schaefer EJ. Effect of gender, age, and lipid status on low density lipoprotein subfraction distribution. Results from the Framingham Offspring Study. Arteriosclerosis. 1987;7(5):483–90. . 3675308 10.1161/01.atv.7.5.483

[67] Davis CE, Williams DH, Oganov RG, Tao SC, Rywik SL, Stein Y, et al Sex difference in high density lipoprotein cholesterol in six countries. American journal of epidemiology. 1996;143(11):1100–6. . 8633598 10.1093/oxfordjournals.aje.a008686

[68] Mascitelli L, Pezzetta F. High-density lipoprotein cholesterol and sex difference in coronary heart disease risk. The American journal of medicine. 2006;119(5):e17 10.1016/j.amjmed.2005.10.044 . 16651038

[69] Abbott RD, Garrison RJ, Wilson PW, Epstein FH, Castelli WP, Feinleib M, et al Joint distribution of lipoprotein cholesterol classes. The Framingham study. Arteriosclerosis. 1983;3(3):260–72. . 6573877 10.1161/01.atv.3.3.260

[70] Weijenberg MP, Feskens EJ, Kromhout D. Age-related changes in total and high-density-lipoprotein cholesterol in elderly Dutch men. American journal of public health. 1996;86(6):798–803. 8659652 10.2105/ajph.86.6.798 PMC1380397

[71] Ferrara A, Barrett-Connor E, Shan J. Total, LDL, and HDL cholesterol decrease with age in older men and women. The Rancho Bernardo Study 1984–1994. Circulation. 1997;96(1):37–43. . 9236414 10.1161/01.cir.96.1.37

[72] Suc I, Escargueil-Blanc I, Troly M, Salvayre R, Negre-Salvayre A. HDL and ApoA prevent cell death of endothelial cells induced by oxidized LDL. Arteriosclerosis, thrombosis, and vascular biology. 1997;17(10):2158–66. . 9351385 10.1161/01.atv.17.10.2158

[73] Sugano M, Tsuchida K, Makino N. High-density lipoproteins protect endothelial cells from tumor necrosis factor-alpha-induced apoptosis. Biochemical and biophysical research communications. 2000;272(3):872–6. 10.1006/bbrc.2000.2877 . 10860844

[74] Robbesyn F, Garcia V, Auge N, Vieira O, Frisach MF, Salvayre R, et al HDL counterbalance the proinflammatory effect of oxidized LDL by inhibiting intracellular reactive oxygen species rise, proteasome activation, and subsequent NF-kappaB activation in smooth muscle cells. FASEB journal: official publication of the Federation of American Societies for Experimental Biology. 2003;17(6):743–5. 10.1096/fj.02-0240fje . 12586748

[75] Lee CM, Chien CT, Chang PY, Hsieh MY, Jui HY, Liau CS, et al High-density lipoprotein antagonizes oxidized low-density lipoprotein by suppressing oxygen free-radical formation and preserving nitric oxide bioactivity. Atherosclerosis. 2005;183(2):251–8. 10.1016/j.atherosclerosis.2005.03.029 . 16098532

[76] Lin MT, Beal MF. Mitochondrial dysfunction and oxidative stress in neurodegenerative diseases. Nature. 2006;443(7113):787–95. Epub 2006/10/20. 10.1038/nature05292 . 17051205

[77] Flaquer A, Heinzmann A, Rospleszcz S, Mailaparambil B, Dietrich H, Strauch K, et al Association study of mitochondrial genetic polymorphisms in asthmatic children. Mitochondrion. 2014;14:49–53. 10.1016/j.mito.2013.11.002 24270090

