# Supplementary figures and images for: Mitochondrial GWA Analysis of Lipid Profile Identifies Genetic Variants to Be Associated with HDL Cholesterol and Triglyceride Levels
Source: PLoS One. 2015 May 6;10(5):e0126294. doi: 10.1371/journal.pone.0126294 (PMC4422732; doi:10.1371/journal.pone.0126294)

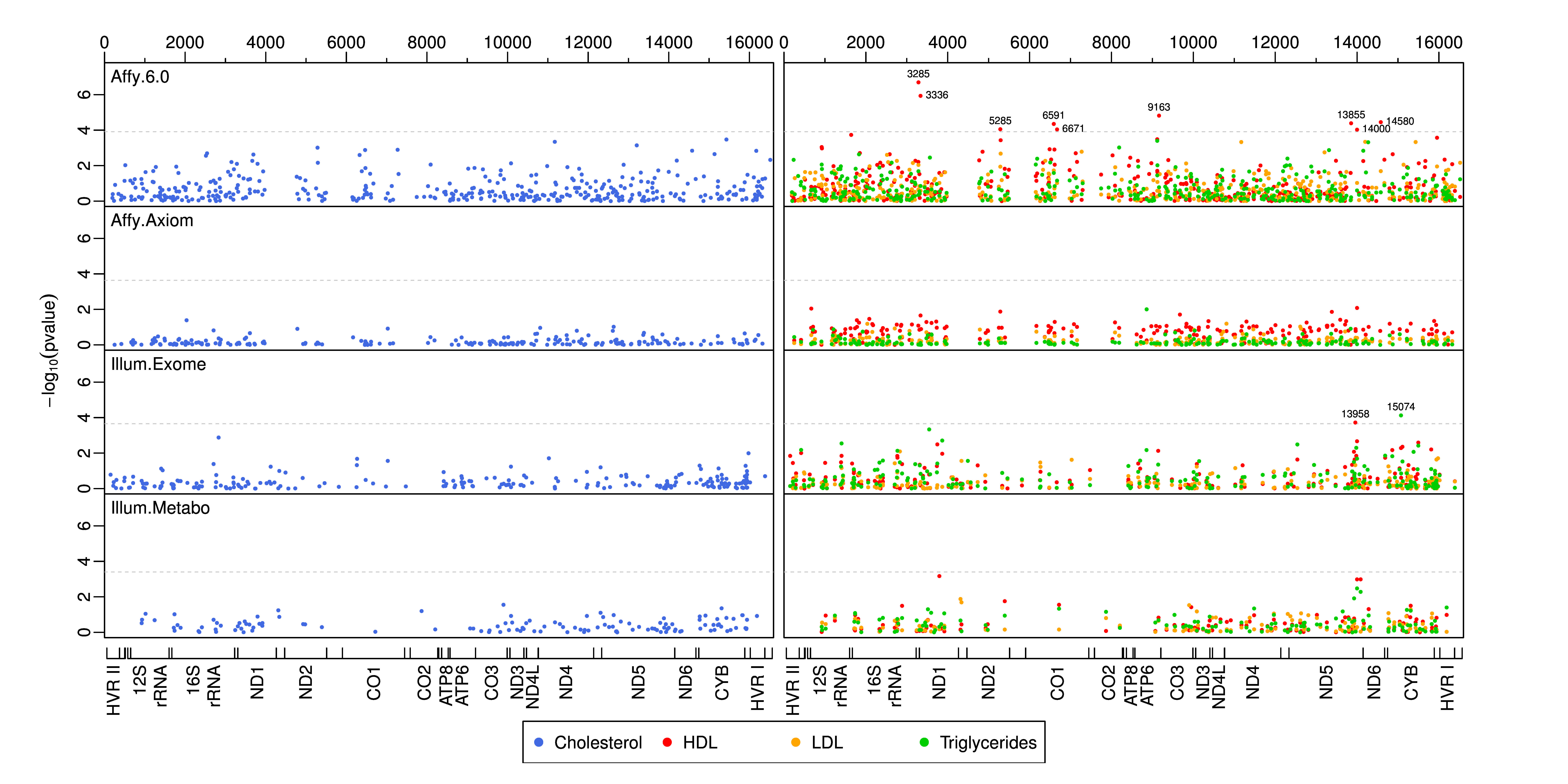

Supplement: S1 Fig — On the y axis, p-values transformed into the negative of the base 10 logarithm, −log10(p-value), are shown. The x-axis represents the mitochondrial genome, displaying the position and relative size of each of the 13 major mitochondrial genes, 12S and 16S rRNAs, hypervariable region 1 (HVR I), hypervariable region 2 (HVR II) as well as the position of the 22 tRNAs (gray). The left side illustrates the results for TC and the right side illustrates the results for cholesterol subtypes (LDL, HDL, and triglycerides). The dashed lines show the critical values of the pointwise significance level corresponding to α = 0.05. (TIFF) [file pone.0126294.s001.tiff]
